# Fleet’s Geode: A Breakthrough Sensor for Real-Time Ambient Seismic Noise Tomography over DtS-IoT

**DOI:** 10.3390/s22218372

**Published:** 2022-11-01

**Authors:** Gerrit Olivier, Braeden Borg, Lawrence Trevor, Boris Combeau, Philippe Dales, Jonathan Gordon, Hemant Chaurasia, Matthew Pearson

**Affiliations:** 1 Fleet Space Technologies, Adelaide 5009, Australia; 2 Centre for Ore Deposits and Earth Sciences, University of Tasmania, Hobart 7005, Australia; 3 Mineral Deposit Research Unit, Department of Earth, Ocean and Atmospheric Sciences, The University of British Columbia, Vancouver, BC V6T 1Z4, Canada; 4 Philippe Dales Software Consulting Ltd., Vancouver, BC V6T 1Z4, Canada

**Keywords:** seismic tomography, seismic sensors, instrumentation, passive seismic, internet of things

## Abstract

As most of the outcropping and shallow mineral deposits have been found, new technology is imperative to finding the hidden critical mineral deposits required to transition to renewable energy. One such new technique, called ambient seismic noise tomography, has shown promise in recent years as a low-cost, low environmental impact method that can image under cover and at depth. Wireless and compact nodal seismic technology has been instrumental to enable industry applications of ambient noise tomography, but these devices are designed for the active seismic reflection method and do not have the required sensitivity at low frequencies for ambient noise tomography, and real-time data transmission in remote locations requires significant infrastructure to be installed. In this paper, we show the development and testing of the Geode—a real-time seismic node purpose-built by Fleet Space Technologies for ambient seismic noise tomography on exploration scales. We discuss the key differences between current nodal technology and the Geode and show results of a field trial where the performance of the Geode is compared with a commercially popular nodal geophone. The use of a 2 Hz high sensitivity geophone and low noise digitiser results in an instrument noise floor that is more than 30 dB lower below 5 Hz than nodes that are commonly used in the industry. The increased sensitivity results in signal-to-noise ratios in the cross-correlation functions in the field trial that are more than double that of commercially available nodal geophone at low frequencies. When considering the full bandwidth of retrievable correlations in our study, using the Geode would reduce the required recording time from 75 h to 32 h to achieve an average signal-to-noise ratio in the cross-correlation functions of 10. We also discuss the integration of a real-time direct-to-satellite Internet of Things (DtS-IoT) modem in the Geode, which, together with edge processing of seismic data directly on the Geode, enables us to image the subsurface in real-time. During the field trial, the Geodes successfully transmitted more than 90% of the available preprocessed data packets. The Geode is compact enough so that several devices can be carried and installed by one field technician, whilst the array of stations do not require a base station to transmit data to the cloud for further processing. We believe this is the future of passive seismic surveys and will result in faster and more dynamic seismic imaging capabilities analogous to the medical imaging community, increasing the pace at which new mineral deposits are discovered.

## 1. Introduction

Seismic data acquisition hardware has evolved tremendously over the last 50 years, and advancements in microprocessors, data storage and Li-Ion batteries have significantly accelerated progress in the past decade [1]. The active seismic reflection industry has moved away from cabled systems to wireless, light-weight, battery-powered and blind nodal systems. The advantages of these blind nodal systems are reductions in field crew sizes, faster acquisition, easier deployment in difficult terrains or arrays with complex geometries and smaller environmental footprint, all driving down the cost of active seismic surveys [2].

At the same time that the active seismic reflection industry experienced this rapid evolution in seismic hardware, there has also been a methodological evolution in the passive seismology community through the wide adoption of ambient seismic noise surface wave tomography. The method utilises faint background vibrations from natural and anthropogenic sources to construct estimates of the seismic Green’s function between station pairs, which effectively turns each receiver into a virtual active source that can be used to image or monitor the subsurface [3]. Although the method was conceived decades ago [4,5], computing cross-correlations on long time series data for multiple station pairs is computationally expensive and only became feasible in the last 20 years.

The combination of ambient seismic noise tomography and nodal seismic stations has led to a so-called boom in boomless seismology in recent years [6]. The seismology community has successfully used ambient noise tomography and nodal seismic stations to image the subsurface to delineate mineral deposits, hydrocarbons, geothermal resources, fault zones, volcanoes and geotechnical structures [7,8,9,10,11,12,13,14,15].

The ease-of-use and low cost of nodal seismic acquisition technology has undeniably accelerated the use of ambient noise tomography for small scale and industry applications. However, these devices were designed for active seismic data acquisition. Ambient noise tomography typically utilises faint, low frequency surface waves compared to the active seismic industry that uses energetic and high frequency P-waves from active sources. As a result, the central frequency and sensitivity of the geophones along with the sampling rates and sensitivity of the acquisition hardware used in these nodes are not ideal for ambient noise tomography. Several researchers have demonstrated that these “high” frequency geophones can record teleseismic earthquakes well below their central frequencies [16,17], but research into improving passive seismic data recorded by nodal seismic stations has been focused on improving installation, e.g., coupling and acoustic insulation [18] and not on the design of the nodes themselves.

Another drawback of ambient noise tomography with blind nodal technology is the long turnaround time from data acquisition to 3D model, since the seismic data can only be accessed after harvesting. Although some blind nodal systems offer real-time wireless quality control or real-time data transmission to a central site [2,19,20], the requirements for ambient seismic noise tomography are considerably different to active seismic imaging from an acquisition time, sampling rate and bandwidth perspective. The low data rates for ambient noise tomography lends itself well to long-range low-power Internet of Things (IoT) networks. In recent years there have been some efforts to develop real-time seismic nodes for ambient noise tomography using IoT sensor networks [21,22,23], but these either require a base station with line-of-sight to each node (similar to current real-time nodes) or narrowband IoT (NB-IoT) coverage for each node to transmit its data to the cloud. These limitations severely impact the utility of these devices in remote regions with limited or no cellular coverage and moderate topography variations that make line-of-sight difficult to establish.

In this paper, we discuss the development and testing of the Geode: a real-time, satellite-enabled seismic node specifically designed for mineral exploration in remote settings. The engineering challenge we tackled was to design a device that is compact enough so that several devices can be carried and installed by one field technician, whilst the array of stations do not require a base station to transmit data to the cloud for further processing. We focus our discussion on the three key improvements that will enable scalability: (1) Using a lower frequency and higher sensitivity geophone, (2) conducting pre-processing on the device (edge processing) and (3) transmitting the pre-processed data to the cloud in real-time using a built-in DtS-IoT modem. We show results from a field trial where the performance of the Geode was compared to a popular nodal seismic station and we discuss the implications of real-time data for remote ambient seismic noise tomography for mineral exploration.

## 2. Materials and Methods

Our goal with the development of the Geode was to make a real-time node for ambient seismic noise tomography at mineral exploration scales. The two areas we believed could be improved were: (1) enabling nodes to transmit data in real-time from anywhere on Earth whilst maintaining a compact form factor, and (2) improving the sensitivity and frequency response of the device to enable ambient noise tomography in remote locations.

### 2.1. Direct-to-Satellite Real-Time Nodes

The relatively low sampling rates (typically 10–50 Hz) and long acquisition times of ambient noise tomography, along with remote locations of mineral exploration, lends itself to low-power wide-area networks (LPWAN). By utilising these low-power networks, the compact form factor of nodal seismic stations can be conserved while still providing real-time data. Indeed in recent years there have been some efforts to develop real-time seismic nodes for ambient noise tomography using IoT sensor networks [21,22,23]. Sepulveda et al. [23] argue that a hub-and-spoke topology, where the network of sensors transmits pre-processed data to a base station, which in turn uploads the data to the cloud using wireless broadband communication (e.g., LTE), is suitable for real-time ambient noise tomography. Satellite communications, on the other hand, provide a more cost-effective alternative compared to other terrestrial solutions and can enable global connectivity [24]. Therefore one can utilise a broadband satellite internet connection (e.g., Starlink by SpaceX) at the base station in areas where no cellular coverage is available. The downside of this approach is that at least one base station needs to be erected with significant height to achieve line-of-sight to each node, while the base station itself has significant power consumption requirements. Both these requirements make the use of a base station impractical and restrictive. Jamali-Rad et al. [22] on the other hand showed that using narrowband internet of things (NB-IoT), an LPWAN radio technology, to directly transmit the pre-processed data to the cloud is a more agile solution that does not depend on a base station. The limitation of this approach is the requirement for cellular (IoT) coverage at each station, which may not be the case in remote and isolated regions where mineral exploration is often conducted.

**Figure 1:**
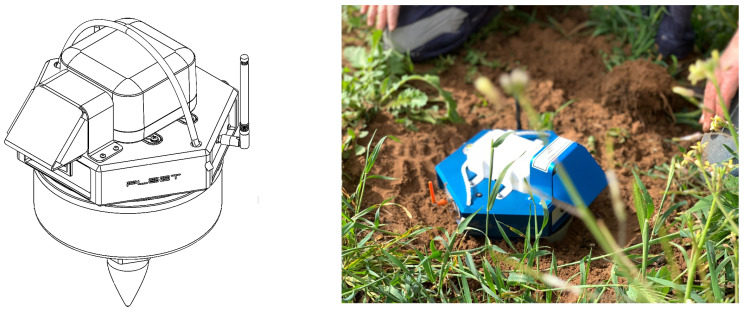
Schematic of the Geode design (**left**) along with a picture of an installed Geode (**right**).

IoT can be described as a system of intelligent computing devices that transfers data over a network without human interaction [25]. In recent years a new type of IoT connectivity has emerged that utilises low-Earth orbit satellite constellations to provide global connectivity: Direct-to-Satellite IoT (DtS-IoT, see Fraire et al. [26] for a review). This solution is ideal for ambient seismic noise tomography stations, as it enables us to conduct ambient noise tomography at low cost anywhere on Earth with compact and agile devices that do not require a base station. A schematic of the design of the Geode is shown in Figure 1 alongside a picture of the Geode installed in the field. The components contained in the Geode (from top to bottom) are the satellite modem, battery pack, enclosure housing the electronics and sensor digitiser, single-component geophone element and spike. To enable the use of DtS-IoT, we reduce the data volumes by performing data pre-processing (or edge computing) directly on the Geode microcontroller (similar to [21,22,23]). It is important to note that the utilisation of edge-processing is main differentiating factor between IoT-enabled seismic devices and conventional real-time seismic stations that utilise cellular or satellite telemetry. The Geode also contains a LoRa antenna for in-field quality control and diagnostics. As currently configured the Geode weighs just under 9 kg and has a battery life of approximately 40 days, which fulfils our design requirements. The size and weight of the Geode can be reduced by either designing for a shorter battery life or building a modem with lower power consumption, which we are actively working on.

### 2.2. Instrument Sensitivity

Modern nodal geophones typically utilise either a 5 Hz or 10 Hz high-sensitivity geophone along with a low-noise digitiser with built in programmable gain [20]. The choice of geophone element (and seismic sensor more broadly) is dictated by the frequency and the amplitude of the signal you wish to record.

**Figure 2:**
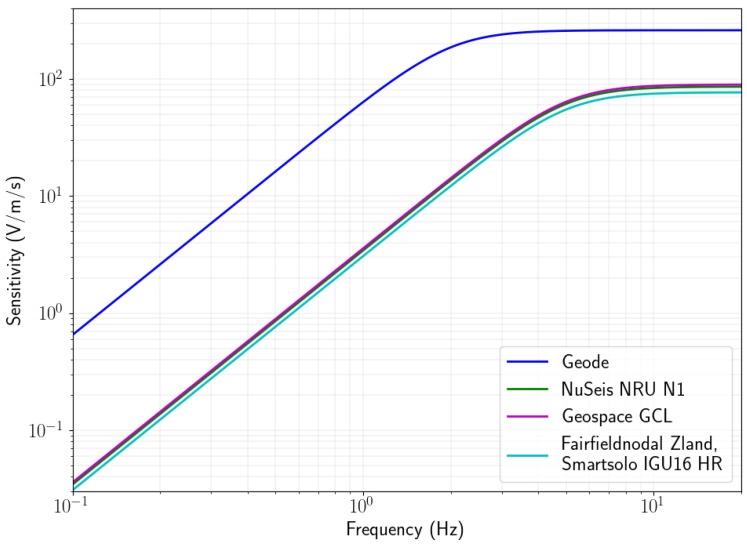
Instrument response function for geophones used in several popular nodal geophones compared to the geophone used in the Geode.

Although the central frequency of the geophone plays an important role in the frequencies one can retrieve, some authors have reported that one can record signals as low as 0.1 Hz from teleseismic earthquakes with 5 Hz geophones, even though Figure 2 indicates that the sensitivity of these geophones are more than 1000 times less than 5 Hz at these frequencies [16,17]. In mineral exploration, we typically use frequencies between 0.1 and 10 Hz for ambient seismic noise surface wave tomography. Theoretically one can use a lower frequency geophone element with higher sensitivity to improve the retrieval of low frequency ambient noise [27], however we did not find any studies on the effects of higher sensitivity and lower frequency geophone elements on the retrieval of cross-correlation functions and the impact on signal-to-noise ratios. Some of the nodal seismic devices allow for the use of an external sensor (e.g., the Nuseis NRU KCK), which could be used with an external sensors such as the SeisTech ST-2A 2 Hz Geophone with KCK connector for further research into the utility of low frequency nodes for ambient noise tomography. Alternatively, some manufacturers have designed nodes with circuits that electronically extend geophone elements to a wider bandwidth and higher sensitivity (e.g., the SmartSolo IGU-BD3C-5).

To ensure the highest possible sensitivity without compromising the compact design of the Geode, we use a 2 Hz geophone with sensitivity of 260 V/m/s. The benefits of this geophone element for ambient noise tomography are also discussed in [27]. A comparison of the instrument response functions for this geophone compared to those used in popular nodal geophones is shown in Figure 2. The geophone is approximately three times more sensitive at frequencies above 5 Hz and approximately 20 times more sensitive at 0.1 Hz.

The sensitivity of the geophone is only part of the overall noise floor of the instrument. To calculate the noise floor of the device we need to divide the effective input noise of the device with the sensitivity of the geophone. The effective input noise is a combination of the geophone noise (Johnson noise) and the input noise of the preamplifier and analogue to digital converter [28]. The effective input noise is also reduced by increasing the gain of the device and/or lowering the sampling rate [28]. Therefore it is important to select the highest gain and lowest sampling rate appropriate for the frequencies one intends to record.

**Figure 3:**
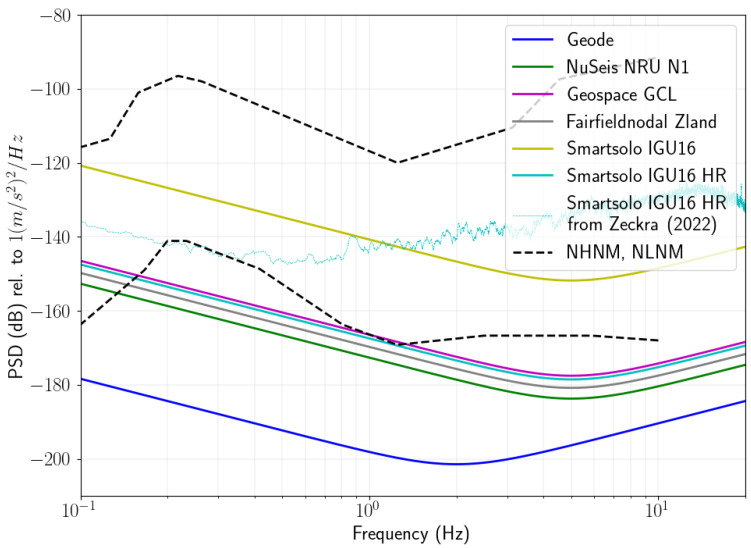
Theoretical self-noise for popular nodal geophones compared to the Geode and the measured self-noise from [16].

In Figure 3, we show a comparison of the theoretical noise floor of several popular nodes compared to that of the Geode. For these graphs we used the specifications from the suppliers to calculate the theoretical self-noise at the lowest programmable sampling rate and the highest gain along with the global lower noise model (NLNM) [29]. The figure also includes the measured self-noise of the Smartsolo IGU16 HR node from Zeckra et al. [16] using the method from Sleeman et al. [30] for the same recording parameters. Although the theoretical noise floor of the traditional nodal geophones is below the NLNM for much of the frequency band of interest, we see that the measured self-noise for the Smartsolo IGU16 HR 5 Hz is higher than the theoretical noise floor (between 10 dB and 45 dB higher) and in fact above the NLNM for nearly the entire frequency band of interest. The difference between the theoretical and actual noise floor is to be expected, since the theoretical calculation does not take some secondary effects, such as electromagnetic interference, into account. Additionally, it was assumed that the equivalent input noise of the device is frequency independent since the value is typically not reported for different frequencies by manufacturers. Possible other causes for the higher measured noise floor may be the effect of installation tilt on the geophone sensitivity.

Since resource exploration is often done in very remote regions where seismic noise levels can be close to the NLNM, it is possible that no useful cross correlation signals can be retrieved between pairs of seismic stations which will in turn mean that ambient noise tomography can not be applied in the appropriate frequency band. In Figure 3, we see that the choice of the geophone, digitiser and acquisition settings for the Geode result in a theoretical self noise that is between 16 and 35 dB below the NLNM between 0.1 and 10 Hz. Even if the theoretical self-noise underestimates the true self noise as discussed above, the design of the Geode reduces the likelihood that ambient noise levels are below the instrument noise floor and thereby significantly increases the probability of producing cross-correlation functions with higher signal-to-noise levels.

The noise level itself is not the only consideration, because we need to record common seismic noise in pairs of receivers to get meaningful correlation. In other words even if both instruments record seismic noise above their self noise, at high frequencies local sources might contribute to the noise recorded in one station, but be well below the noise floor of the other due to attenuation. Since attenuation is very hard to model, the only way to know whether device sensitivity can improve our chances of recording common noise in remote locations is to make field measurements. In the following section we show the results of a field trial in a remote part of South Australia.

## 3. Results

**Figure 4:**
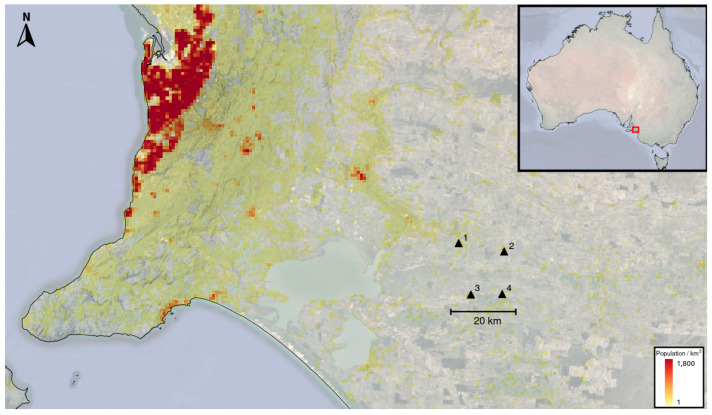
Location of the 4 sites where the Geodes were tested. Each site consisted of a buried Geode with a collocated Nuseis node. The sites were all situated in an area with low population density.

We recorded ambient seismic noise for seven days and compared the resulting correlations functions from four real-time Geodes with four collocated blind Nuseis NRU N1 5 Hz nodes. The location of the field trial was chosen for two reasons: (1) the area is remote with a low population density (see Figure 4) and (2) the area is covered by an approximately 100 m thick layer of unconsolidated sediments (predominantly sand and clay). The low population density means that anthropogenic noise is low, whilst the layer of unconsolidated sediments attenuates seismic energy rapidly. Both these factors are expected to result in low ambient seismic noise levels at higher frequencies (above 1 Hz).

In order to achieve the lowest instrument self-noise possible, we utilised the lowest sampling rate (250 Hz) and highest gain setting (42 dB) for the Nuseis NRU N1 nodes. Care was taken to ensure that both the Geode and Nuseis nodes were installed as close as possible to vertical position, and the tilt angles were checked again during retrieval. During the experiment the Geodes transmitted more than 90% of the preprocessed data packets. The missing data packets were not received due to telemetry outages, but we aim to address this in future with appropriate buffering on the Geodes. To compute the correlation functions, we used the approach of [31] with window length of 20 min and whitening frequency band of 0.05–5 Hz and one-bit normalisation before correlation. If whitening is performed prior to one-bit normalisation, restitution of the instrument response is redundant.

**Figure 5:**
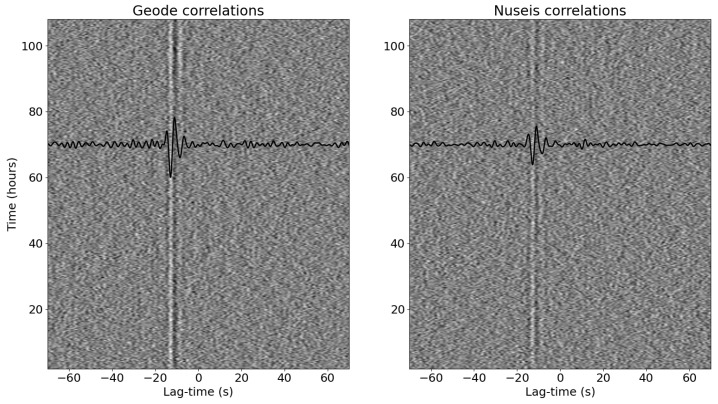
Comparison of the correlation functions for the Geodes compared to the Nuseis nodes. The waterfall plot shows the correlation function for each 20 min segment, while the black line shows the stacked correlation function.

In Figure 5, we show a comparison of the cross correlation functions for each 20 min window for stations 1 and 4, which are approximately 18 km apart, as raster images with the stack of all the correlations in black. Identical processing was used for the two pairs of devices. We can see that the 20 min correlations for the Geodes show a clearer arrival at −12 s lag time for certain time periods (e.g., from hours 90–110) than the Nuseis nodes. The stack of the correlations also show a clearer peak for the Geodes compared to the Nuseis nodes. The stacked correlations show a peak at −12 s lag-time, which likely indicates that the dominant noise source is the southern Indian Ocean to the south of station 4. The missing data packets from the Geodes due to telemetry outages appeared to have minimal effect on the resulting correlations.

**Figure 6:**
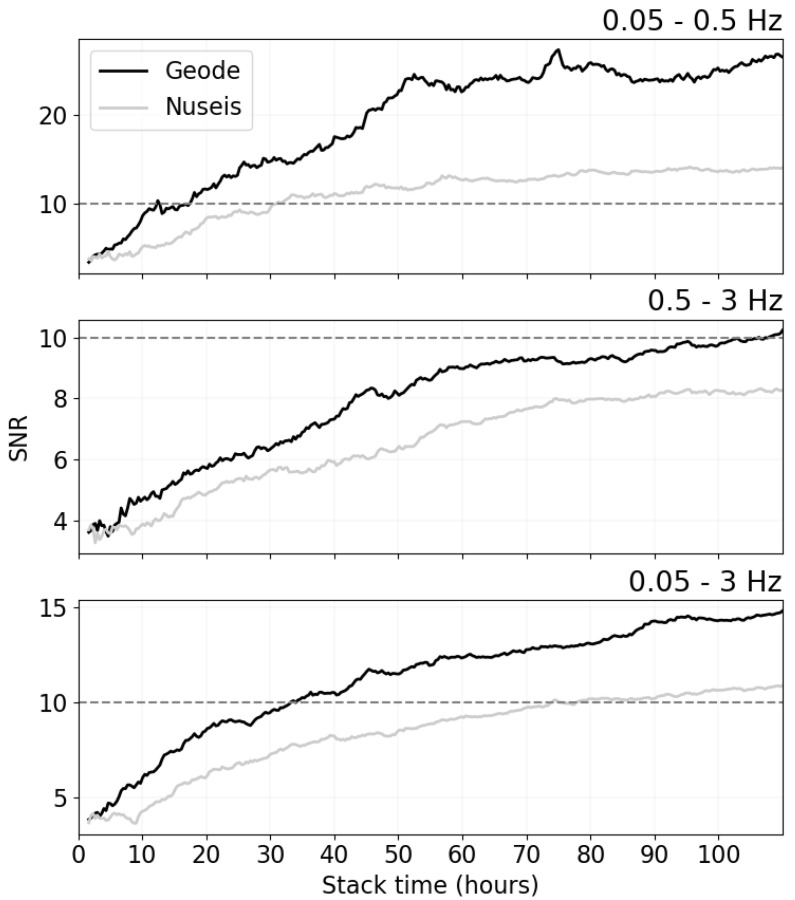
Comparison of the SNR of the average of the 6 Geode correlation functions (black) and those of the Nuseis correlation functions (grey) for three different frequency bands. The black dashed line indicates an SNR of 10, which is the threshold for making dispersion measurements.

To quantitatively compare the performance of these devices, we investigate the signal-to-noise ratio (SNR) of the resulting cross correlation functions between pairs of stations. A lower SNR results in higher uncertainty in phase velocity measurements and ultimately lower resolution in the final tomographic images [32]. We define the SNR as the ratio of the peak amplitude within the Rayleigh wave arrival window divided by the root mean square of the trailing tail of the correlation function, following the convention of Bensen et al. [31]. In Figure 6, we show the average SNR for all station pairs for three frequency bands, namely 0.05–0.5 Hz (low), 0.5–3.0 Hz (high) and 0.05–3.0 Hz (full). We see that the SNR is higher for the Geode correlations for all three bands. As expected from Figure 2 and Figure 3, the improvement is the greatest for the low frequency band. However, the improvement for the other frequency bands are also remarkable. We did not retrieve clear peaks in the correlation functions for either device above 3 Hz, likely due to the large inter station distance relative to wavelength and the attenuating cover layer.

Most ambient noise tomography studies only make surface wave dispersion measurements on cross-correlation functions with SNR of 10 or higher [32,33]. Bearing this in mind, the implications of the increased SNR for the Geode compared to conventional nodal devices are significant. In this particular case, the required recording time is reduced from approximately 75 h to less than 35 h when considering the entire measurable frequency band. We also note that the SNR for the Nuseis nodes in the high frequency band (0.5–3 Hz) only reaches an SNR of just over 8 during the 110 h recording period.

From the instrument response curves and self-noise in Figure 2 and Figure 3, we expect the SNR in correlation functions from Geodes to always be better than (or at least as good as) the SNR from conventional nodal geophones. The improvement will vary from case to case as frequency range, inter station distance, geological conditions and noise levels would all play a part in the recorded data. In general we expect the difference to be the greatest in remote regions far from anthropogenic noise. On the other hand, one would expect a minimal difference for surveys conducted in urban environments with short interstation distances where the frequency and amplitude of the ambient noise field would likely be significantly higher.

The field test showed that the design of the Geode will result in significant improvements of the SNR of the ambient seismic noise cross correlation functions compared to conventional blind nodes. This is particularly the case in remote regions where mineral exploration is often conducted.

## 4. Discussion: Real-Time Geodes and Dynamic Arrays

In this paper, we discussed the development of a seismic node that transmits data in real-time directly to satellite IoT networks and that is significantly more sensitive than conventional nodal geophones. We believe that the shorter acquisition times and dynamic arrays from these innovations will have significant impacts on the scalability of ambient seismic noise tomography for mineral exploration. Speed is crucial for mineral exploration, as one has to image large areas with high resolution at low cost to define targets for further exploration. Dynamic arrays enable scalable and adaptable resolution based on initial imaging results.

Currently, the length of data acquisition is often determined by battery life of the device as there is no way for seismologists to know when enough data has been recorded with blind acquisition systems. The only way to determine the required data acquisition time for blind nodal systems is to conduct a truncated test (e.g., noise test) and assume that the noise conditions remain the same for the full-scale deployment, or to harvest some data in the field on a regular basis to assess the SNR of the correlation functions. These options add significant logistical complexity and/or survey time. The SNRs of the correlation functions, which ultimately dictate the accuracy of the results, are dependent on the seismic noise levels and geological conditions along with the instrument sensitivity as discussed in a previous section. With real-time data acquisition, it is possible to dynamically reduce (or increase) the acquisition time based on the SNR of the correlation functions to ensure that adequate data is recorded for imaging purposes in the shortest possible time. The results of our field trial here can serve as an example, where the Geodes retrieved correlation functions with average SNR higher than 10 in less than two days for the full frequency band (0.05–3 Hz), which means that the survey could have been stopped after two days, whereas a survey conducted with conventional nodes might have been more than 20 days.

Speed is not the only benefit. The idea of a dynamic array comes from the medical imaging field, where ultrasound technicians would for instance move the transducer whilst viewing the monitor in real-time to get the best view of the target [34]. In ambient seismic noise tomography, by moving seismic stations to areas that show promising geophysical features, we can reduce the acquisition time and cost per km${}^{2}$ significantly by not “wasting” resolution in barren areas. This idea is not novel in the exploration industry, as “filling-in” or “in-fill” campaigns are routinely conducted after initial coarse scale surveys for geophysical methods, soil sampling, and exploration drilling [35,36]. Dynamic arrays can also be used with C3, a method that can “bridge” sequential seismic arrays to increase resolution of the tomographic image by fixing the location of some stations whilst moving the other [37].

## 5. Conclusions

Over the last 10 years technological (nodes) and methodological (ambient seismic noise tomography) advancements have led to a boom in boomless seismology. Although most studies have been conducted in academia, the benefits of this method to the mineral exploration industry have become clear in the last few years. The main drawbacks of traditional methods are the long acquisition and turnaround times. To address these issues we developed a seismic node purpose-built for ambient seismic noise tomography—the Geode. The Geode utilises a high-sensitivity 2 Hz geophone element with a low-noise digitiser. This results in a self-noise floor that is more than 30 dB lower below 5 Hz than nodes that are commonly used in industry. We demonstrated the benefits of the increased sensitivity of the device in a field trial where four Geodes recorded data for approximately five days alongside collocated Nuseis NRU N1 nodes in a remote location in South Australia. The resulting cross-correlation functions from the Geodes have significantly higher SNR, which results in 2× shorter recording times and a lower uncertainty in dispersion measurements, and ultimately in the 3D models that they produce. The Geode also has a built-in satellite modem that transmits pre-processed data in near real-time to low-Earth orbit satellites via DtS-IoT. Real-time data acquisition will greatly increase the speed at which an area can be imaged and will enable the use of dynamic imaging arrays, which will increase resolution and improve targeting for mineral exploration. Our future research will involve reducing the power consumption and size of the Geode, whilst extending the capabilities by recording multiple sensing components and transmitting other data via DtS-IoT (e.g., seismic triggers, H/V spectra).

## 6. Patents

Patent application 2022209325 filed 28 July 2022. Title: “Satellite-enabled node for ambient noise tomography”. 

## Data Availability

Data available on request due to restrictions.
